# Incorporating a Fresh Mixed Annual Ryegrass and Berseem Clover Forage Into the Winter Diet of Dairy Cows Resulted in Reduced Milk Yield, but Reduced Nitrogen Excretion and Reduced Methane Yield

**DOI:** 10.3389/fvets.2020.576944

**Published:** 2020-11-20

**Authors:** Daniel Enriquez-Hidalgo, Dayane Lemos Teixeira, Luiz Carlos Pinheiro Machado Filho, Deirdre Hennessy, Paula Toro-Mujica, Shaun Richard Owen Williams, Fabiellen Cristina Pereira

**Affiliations:** ^1^Bristol Veterinary School, University of Bristol, Langford, United Kingdom; ^2^Facultad de Agronomía e Ingeniería Forestal, Pontificia Universidad Católica, Santiago, Chile; ^3^Rothamsted Research, Sustainable Agriculture Sciences, Okehampton, United Kingdom; ^4^Instituto de Ciencias Agroalimentarias, Animales y Ambientales (ICA3), Universidad de O'Higgins, San Fernando, Chile; ^5^Laboratorio de Etologia Aplicada, Universidade Federal de Santa Catarina, Florianópolis, Brazil; ^6^Teagasc, Animal & Grassland Research and Innovation Centre, Moorepark, Fermoy, Ireland; ^7^Agriculture Victoria Research, Ellinbank, VIC, Australia

**Keywords:** mixed herbage, *Trifolium alexandrinum*, *Lolium multiflorum*, total mixed ration, dairy cattle, enteric methane, milk production, milk quality

## Abstract

The winter diet of dairy cows in Mediterranean climate regions is usually a total mixed ration with a base of conserved summer crops such as corn silage and alfalfa hay. However, there is increased labor and financial cost related to this kind of feeding, which could be reduced if fresh forages were used in place of some of the conserved forage in the cow diet. The objective of our study was to evaluate the effect of including fresh mixed annual ryegrass and berseem clover into the diet of dairy cows on milk, nitrogen utilization, and methane emission. Twenty-four lactating dairy cows were split into two groups and offered either a diet similar to that usually offered to the cows (CON) or one where a mixture of fresh annual ryegrass and berseem clover was used to partially substitute the corn silage and alfalfa hay in the diet (MIX). Milk yield was recorded automatically, and methane emissions were estimated using the SF_6_ tracer technique. The MIX diet had lower crude protein concentration (148 vs. 170 g/kg DM) but higher DM digestibility (81.6 vs. 78.6%) than the CON diet. Compared to the cows offered the CON diet, milk yield was reduced when cows were fed the MIX diet (36.4 vs. 31.9 kg/d), but methane emissions (381 vs. 332 g/d) and nitrogen excretion were also reduced (238 vs. 180 g/d). Nitrogen use efficiency was unaffected (30.8%). In addition, milk from cows fed the MIX diet had a fatty acid profile considered to be more beneficial to human health than that of the milk from cows fed the CON diet. Increasing the protein concentration in the MIX diet, either by direct supplementation or increasing the proportion of legume in the mixed herbage, could overcome the reduction on milk and positively affect methane emission and N use efficiency.

## Introduction

The winter diet of dairy cows in Mediterranean climate regions is usually a total mixed ration (TMR) with a base of conserved summer crops such as corn (*Zea mays* L.) silage and alfalfa (*Medicago sativa* L.) hay. However, this conserving and storage come at a financial cost related to additional machinery usage, storage facilities investment, and storage consumables. This cost could be reduced if fresh forages were used in place of some of the conserved forage in the TMR. Including fresh forage in the diet of dairy cows has been reported to have no effect on milk composition (1, 2) or milk yield (MY) (1, 3, 4). However, there is little information regarding the effect of mixed-sward forages when included in the TMR for dairy cows.

Legumes, with their lower fiber and greater protein concentration and greater digestibility than grasses, have been reported to increase MY and milk protein concentration when incorporated into the diet of dairy cows (5, 6). Legumes have also been reported to change the fatty acid (FA) profile of the milk, increasing the concentration of those FA considered to be beneficial to human health, such as linoleic acid, vaccenic acid, rumenic acid, and n-3 FAs (4–6). A forage legume used in Mediterranean-type climates and some subtropical regions as a fresh winter forage is berseem clover (*Trifolium alexandrinum* L.) (7). As a legume, berseem clover can fix more than 200 kg N/ha/year (8). Additionally, unlike other legume species, berseem clover does not cause bloat in ruminants (9) and therefore can be fed alone. Despite its advantages, berseem clover is commonly grown in mixed swards with annual ryegrass (*Lolium multiflorum* Lam) to optimize forage production (8, 10). However, the effect of including fresh berseem clover, either singly or as part of a mixed forage sward, in the diet of dairy cows on metabolic processes, MY, and milk quality is poorly documented.

Enteric methane emissions of ruminants are reported to increase with increasing proportion of forage in the diet (11). However, this relationship between methane and diet is influenced by forage quality. High-quality forage, with high protein and low fiber content, can result in reductions in methane emissions similar to the use of grains in the diets of dairy cows (12), due to changes on rumen fermentation, such as reduced rumen pH, low acetate/propionate ratio, high NH_3_-N concentration, and high rate of passage (3). Incorporating fresh, high quality, grass into the diet of dairy cows has been reported to reduce their methane emissions (2). Other high-quality forages, such as legumes, have also been reported to reduce methane yield [g CH_4_/kg dry matter intake (DMI)] when incorporated into the diet of dairy cows, due to increased feed intakes (13). Because of the activity of nitrogen (N)-fixing, legumes also have the additional advantage of having secondary compounds (14). These secondary compounds (e.g., tannins, alkaloids) can contribute to the reduction of methane emissions (15), such as reported by (16) where the incubation of 130 to 185 g hydrolysable tannins/kg DM suppressed the total population of methanogens *in vitro* by on average 12%. Legumes also improve energy and N use efficiency offering high N intake with high milk protein yield and reduced N loss (17). Thus, incorporating legumes into the diet of dairy cows should have milk production and environmental benefits.

The objective of this study was to evaluate the effect of fresh mixed annual ryegrass and berseem clover inclusion into the TMR of dairy cows during the winter period on MY, milk quality and FA profile, nitrogen utilization, and methane emissions. We hypothesized that replacing around 40% of the alfalfa hay and corn silage in the TMR of dairy cows with a fresh forage mixture (annual ryegrass and berseem clover) would (1) sustain similar MY, (2) reduce emissions of enteric methane, and (3) increase the efficiency of use of dietary nitrogen.

## Materials and Methods

**Figure 1:**
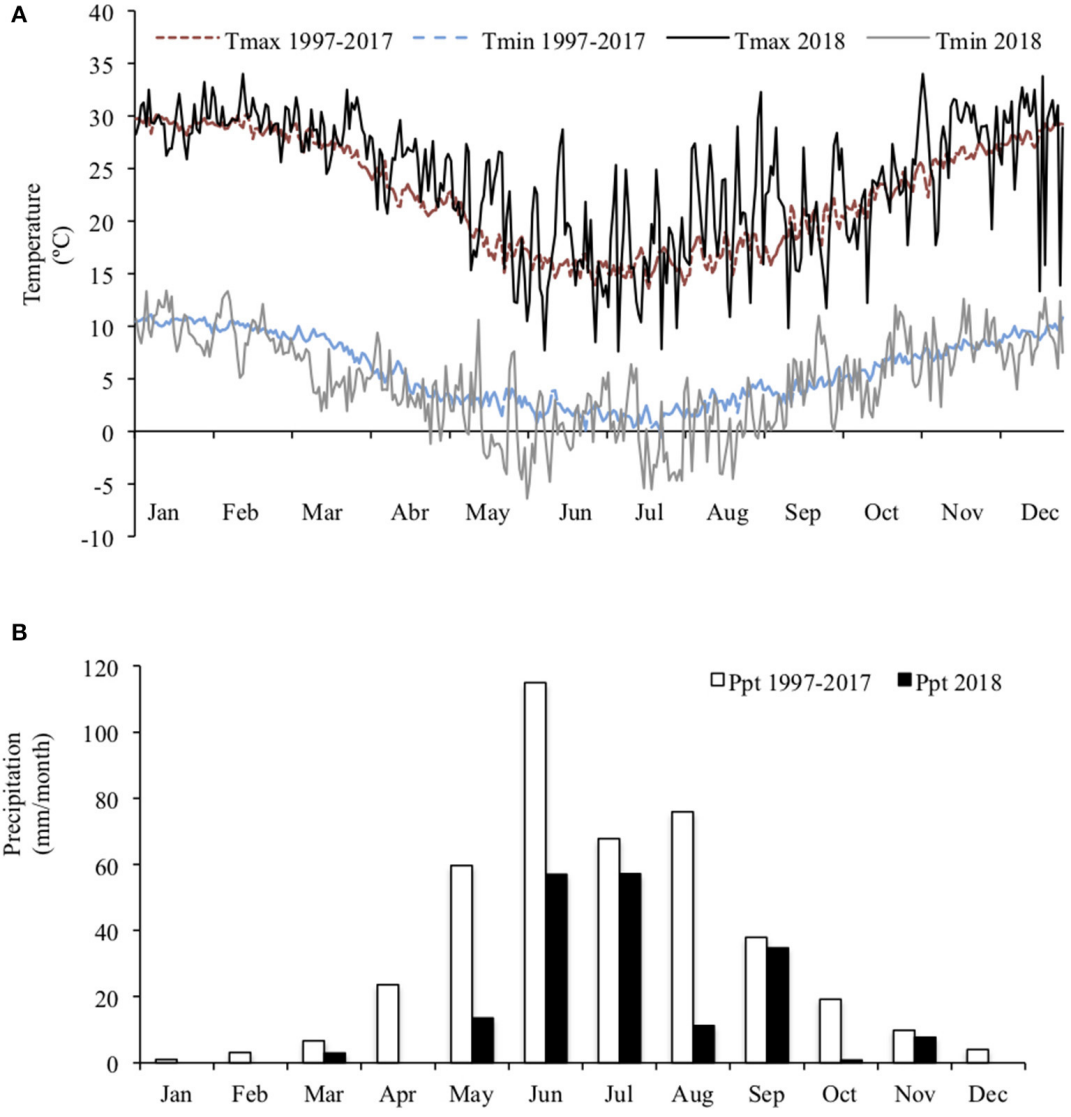
Mean maximum and minimum daily temperature **(A)** and total monthly rainfall **(B)** during the experimental year 2018 and averaged over the last 30 years at the experimental site.

The experiment was conducted at the farm of the Pontificia Universidad Católica de Chile, located in Pirque, (33°40′S; 70°36′W), from August to October 2018. The climate in this region is characterized as Csb (Mediterranean with hot summers), according to the Köppen climate classification. The average annual rainfall is 424 mm, concentrated from May to September and the average temperature is 14.2°C (Figure 1). The soil of the farm is classified as a Ultic Haploxerolls (Mollisols), consisting of flat land with deep alluvial loam soils.

Twenty-four lactating dairy cows from a herd of 220 cows were split into two groups according to their days in milk (DIM; 0–200 or >200) and assigned to 12 blocks according to breed (Holstein-Freisan and Montbeliard), lactation number [1.6 (0.76)], DIM [222 (84.7) d], and pre-experimental MY [37.8 (4.27) kg/d]. Cows within blocks were randomly allocated to one of two groups. Each group received either (1) a control (CON) diet similar to the diet usually offered to the cows where the forage fraction was made of corn silage and alfalfa hay or (2) an experimental diet (MIX) where a fresh annual ryegrass and berseem clover mixed herbage was used to substitute 40% of the corn silage and alfalfa hay forage fraction of the CON diet. The composition of each diet is shown in Table 1. Diets were formulated to be isocaloric (11.09 MJ/kg) and isonitrogenous [165 g/kg DM crude protein (CP)] according to the cows requirements (18) and chemical composition of ingredients using data from previous years. The diets were offered for 10 weeks. Cows were randomly allocated to individual pens (6.0 × 3.5 m) and received their diets *ad libitum* (regulated to 5% refusals) offered half after the morning and half after the afternoon milkings. A mixer wagon (Euromix II, Kuhn, UK) was used to mix diet ingredients before each feeding. Cows had *ad libitum* access to an individual water trough. Cows were milked three times per day at 7:00 am, 3:00 pm, and 9:00 pm.

**Table 1:** Ingredients of the diets offered to the cows during the experimental weeks: CON diet and experimental diet (MIX).

**(% of DM)**	**CON**	**MIX**
**Diet ingredients**		
Ground corn	16.7	20.2
Wheat bran	14.4	18.2
Canola	23.0	11.2
Corn silage	34.2	25.2
Alfalfa hay	8.4	—
Wheat straw	3.3	—
Annual ryegrass and berseem clover	—	25.2

The forage sward, berseem clover and annual ryegrass mix, was established in February 2018. Soil was prepared with a primary tilling and two secondary tilling before sowing. Sowing was by seed drill with annual ryegrass (cv. Winter Star II, Anasac) at 100 kg/ha and berseem clover (cv. Elisa, Agro-UC) at 120 kg/ha. Fertilizer was applied at sowing at a rate of 66 kg N/ha, 313 kg P_2_O_5_/ha, and 511 kg K_2_O/ha. The sward received three surface irrigations by flood until April, and then water was from natural rainfall only, 174 mm from May until September. The swards were cut in June, and the second cut was used for the purpose of this experiment. The fresh mixed forage was cut every morning to a height of 6 cm above ground level using a front-mount mower (Crop chopper® model 38, New Holland, US) and loader wagon mounted on a tractor (Agrolux 95, Deutz-Fahr, Germany) and then stored outdoors protected from rain and direct sunshine exposure. The alfalfa hay and corn silage were used from the farm forage stocks, from material harvested during the summer of 2017–2018.

### Feeds and Diets

#### Herbage Characteristics

Characteristics of the herbage were estimated twice a week from samples randomly selected in the area to be cut for the following day. Herbage mass was estimated from four 0.25-m^2^ quadrats. Samples were cut to a height of 6 cm above ground level using hand shears (Accu 60, Gardena International GmbH, Ulm, Germany). The fresh sample was weighed, and a 100-g subsample was oven-dried at 105°C for 5 h to estimate dry matter (DM) concentration [method 934.01; (19)].

Botanical composition of the mixed herbage was estimated from a composite herbage sample (1,000 g of herbage) taken from cutting to a height of 6 cm above ground level at least 10 random locations using the hand shears. Three subsamples of 150 g each were randomly taken, and fractions of annual ryegrass, berseem clover, and other species were manually separated. The separated fractions were then oven-dried at 105°C for 5 h to estimate each herbage fraction DM concentration. Neither herbage mass nor botanical composition was estimated in week 8 of the experiment due to the data being lost.

The phenological stages of the annual ryegrass (20) and berseem clover (21) were determined by identifying the phenological stages of 12 plants within four 0.25-m^2^ quadrats.

#### Diet Composition

Dry matter was determined from samples of each diet taken randomly in triplicate (500 g/sample, as fed) three times per week and oven-dried at 105°C for 5 h to quickly estimate DM concentration to adjust the fresh forage inclusion rate. Similarly, feed residuals left by cows from the same treatment were mixed, and three composite samples (300 g) were used to determine DM concentration.

Chemical analysis was performed on diets, and the individual forage ingredient samples collected twice per week. A subsample (300 g/sample, as fed) from each diet was oven-dried at 60°C for 48 h, milled to pass a 1-mm screen and stored for further chemical analysis. The AOAC (19) methods were used to estimate DM (method 2001.12), CP (method 2001.11), ether extract (method 920.39), lignin (method 973.18), and ash (method 942.05) concentrations. The neutral detergent fiber (NDF) and acid detergent fiber (ADF) were determined using the Van Soest (22) method. The cellulose and hemicellulose concentrations were estimated by difference between NDF, ADF, and lignin concentrations. The *in vitro* DM digestibility and *D* value (digestible OM/DM × 100) were estimated using the Goering and Van Soest (23) methods. The metabolic energy (ME) was determined through the equation (*ME* = 0.279 + 0.0325 × *D* value) of Garrido and Mann (24). To determine the N intake, the residuals of TMR offered to the cows were pooled by treatment in the last week of the experiment and then sampled and analyzed for chemical composition as previously stated. The same methodology was used to estimate the chemical composition of herbage, silage, and hay used to prepare the TMR.

Fatty acids were determined from samples of the diets, herbage, silage, and hay collected during weeks 3, 5, 7, and 9. Samples were collected onto ice and stored at −20°C. The frozen samples were lyophilized before a direct transesterification (25) was performed with 1 mL of methylester as internal standard (1 mg/mL of 23:0 methylester, *n-*23-M de Nu-Chek Prep Inc., Elysian, MN, USA). Fatty acids methyl esters (FAMEs) obtained from the samples were purified by thin-layer chromatography (25) prior to gas chromatography (GC) analysis. Analyses of FAME were performed in a GC equipped with a flame ionization detector (GC-2010 Plus; Shimadzu®, Kyoto, Japan). The FAME obtained were analyzed with a 100 m SP-2560 column and the temperature program plateauing at 175°C (26). Hydrogen (99.999% purity, Air Liquide, Valencia, Spain) with a constant flow rate of 1 mL/min was utilized, and the injector and detector ports were set at 250°C. For peak identification purposes, several reference standards were used: #463 and #603, individual FAME (henicosylic acid C21:0, tricosylic acid C23:0, cerotic acid C26:0, montanic acid C28:0), and a conjugated linoleic acid mixture #UC-59M from Nu-Chek Prep (Elysian, MN, USA); 20:3n-9 FAME; positional isomers of linoleic (C 18:2 n-6; CRM47791) and linolenic (C 18:3 n-3; CRM47792) from Sigma–Aldrich; and a bacterial FAME mixture for branched-chain FA identification from Matreya (Pleasant Gap, PA, USA). In addition, the identities of several FAMEs were confirmed by fractionation using silver-ion solid phase extraction technique (26, 27). Identification of FAMEs not included in the standards were performed by retention times and elution orders reported in the literature as detailed in Bravo-Lamas et al. (28).

### Animal Measurements

The amount of feed offered to each cow and their individual residuals were weighed daily to estimate the average DMI. Cow body weight (BW) and body condition score (BCS) were estimated in weeks 3, 4, 7, 8, and 9. A measuring tape was used to measure the cows' thoracic perimeter to estimate their BW through the equation described by Tebug et al. (29). Cow BCS was assessed using a 1 (sub condition) to 5 (super condition) score (30), given to each cow through visual evaluation made by the same trained observer.

#### Milk

Individual cow MY was automatically recorded daily using milk meters (MM15; Delaval, Tumba, Sweden). Milk composition (fat, protein, lactose, and urea) was determined once per week on weeks 3, 5, 7, 9, and 10 from samples taken at two successive morning and afternoon milkings and analyzed via near-infrared spectroscopy (NIRS) using a Milkoscan 203 instrument (DK3400; Foss Electric, Hillerød, Denmark). Milk solids yield (MSY) was calculated as the yield of milk fat plus the yield of milk protein. The energy corrected milk (ECM) was estimated according to the following equation (31):

$$\begin{array}{l} \text{ECM}=[(0.327\times \text{kg of milk})+(12.95\times \text{kg of milk fat})\\ +\;(7.20\times \text{kg of milk protein})]. \end{array}$$

The FA profile of milk from individual cows was estimated on weeks 3, 5, 7, and 9 from one composite sample of the milking samples taken for milk composition and proportionally pooled according to MY. The FA profile was estimated using a GC analyzer (Shimadzu Scientific Instruments AOC-20s, Columbia, MD, USA) equipped with a 100-m column (Rtx column; 100 m × 0.32 mm × 0.20 μm). Fatty acids peaks were identified by using a FAME standard (Supelco 37 Component FAME mix, Bellefonte, PA); reference standards for C18:1 *trans*-11 and C18:1 *cis*-9, *trans*-11 (Nu-Chek Prep Inc., Elysian, MN) methyl esters were used (32). The atherogenic and thrombogenic indices were calculated using the equations of Ulbricht and Southgate (33), with C12:0, C14:0, and C16:0 as atherogenic and C14:0, C16:0, and C18:0 as thrombogenic FA.

#### Methane

Because of equipment availability, collection of gas samples for estimating methane emissions were undertaken over 6 consecutive days in 2 weeks with the cows in each treatment split into two groups. Emissions of the first 12 cows (6 cows from each diet treatment) were estimated on week 7 and of the second group of 12 cows on week 9 of the experiment. The delay between groups was required to enable cleaning and maintenance of gas collection canisters.

The individual methane emissions were estimated using the modified SF_6_ technique (34). Briefly, cows were orally dosed with a previously calibrated permeation tube containing 2.5 g of SF_6_ 7 days before gas sample collection began. The equipment consisted of a leather head halter with a sampling point above the cow's nostrils, and saddle onto cow's back holding the sample collection canisters. One canister collected expired and eructated gases over 24 h; the second canister collected ambient gases. Canisters were evacuated prior to use, and sampling rate was restricted by orifice plates. Canisters were replaced daily after morning milking and once removed; subsamples of gas were transferred from each canister to pre-evacuated vials. Canisters were evacuated and flushed with N_2_ before being used again.

Concentrations of methane and SF_6_ were determined using a GC (Perkin Elmer Clarus 600; Waltham, MA, USA). A Carboxen 1010 plot column (15 m × 0.32-mm ID, Supelco, Sigma–Aldrich, St. Louis, MO, USA) was used to separate methane and its detection was by a flame ionization detector operating at 250°C. An Elite-GC GS Molesieve column (30 m × 0.53-mm ID × 50-μm film thickness, Perkin Elmer, Waltham, MA, USA) was used to separate SF_6_ and its detection was by an electron capture detector operated at 300°C. All samples were analyzed in duplicate.

Concentrations of atmospheric methane and SF_6_ were subtracted from the values obtained from the cow's canisters and individual daily methane emissions were calculated using Equation 2 of Williams et al. (35). Methane was expressed as methane emission (g/d), methane yield (g/kg DMI), and methane intensity (g/kg various milk parameters).

#### Nitrogen Balance

Individual blood, urine, and feces samples were taken in week 10, on the last day of the experiment. Samples were collected in duplicates ~4 h before and after morning feeding according to the protocol of Colmenero and Broderick (36). Blood samples (50 mL/cow) were collected after morning and afternoon milkings via jugular puncture. Blood was directly transferred to tubes containing lithium heparin (BD Vacutainer, Franklin Lakes, NJ, USA) and immediately centrifuged for 15 min at 3,000 g (C-28A; BOECO, Hamburg, Germany) for plasma separation. Plasma was transferred into 1.5 mL Eppendorf tubes then stored at −20°C until analysis. Urine and feces samples were collected either before morning and afternoon milking as voided or after milking by stimulation by stroking the side of the vulva (urine) or rectal grab (feces). Urine samples were acidified with 40 mL of 0.072 N H_2_SO_4_ and stored in 50 mL Falcon tubes at −20°C. Feces were stored frozen at −20°C until processing and analysis. Plasma ureic N and urine N were determined spectrophotometrically using an automated HumaStar 200 (Human GmbH, Germany). Fecal DM concentration was estimated by oven-drying at 60°C for 48 h and milled to pass a 1-mm screen, and then the AOAC (19) method (2001.11) was used to estimate N concentration as previously described. The daily fecal N was estimated by multiplying total daily feces excretion by fecal N concentration and daily urine ureic N by multiplying total daily urine excretion by urine ureic N concentration. Total daily feces and total daily urine excretion were estimated according to the equations of Wilkerson et al. (37):

$$\begin{array}{l} \text{Total daily feces excretion }\left(\text{kg}/\text{d}\right)=-7.6+\left(0.0256\times \text{BW}\right)\\ +\;\left(0.0238\times \text{DIM}\right)+\left(0.918\times \text{MY}\right)-\left(1.077\text{dietaryCP}\right)\\ +\;\left(0.483\times \text{dietaryNDF}\right)\\ \text{Total daily urine excretion }\left(\text{kg}/\text{d}\right)=\text{total daily manure}\\ \text{excretion}-\text{total daily feces excretion}\\ \text{Total daily manure excretion }\left(\text{kg}/\text{d}\right)=-21.94\\ +\;\left(0.0286\times \text{BW}\right)+\left(0.0378\times \text{DIM}\right)+\left(1.0689\times \text{MY}\right)\\ +\;\left(9.67\times \text{dietary CP}\right)+\left(61.4\times \text{Dietary NDF}\right) \end{array}$$

Nitrogen use efficiency was calculated as the percentage of N intake converted into milk protein N.

### Statistical Analysis

Data was analyzed using SAS (SAS Institute, Cary, NC, United States). Chemical composition data (DM, CP, NDF, ADF, and ash concentrations) and the herbage measurements (herbage mass, herbage botanical composition, and plants phenological stage) were analyzed using generalized linear models (GLM procedure). The model included diet type, week, and their interaction, as fixed effects. Week was used as repeated measure and each sample taken within week was considered as experimental unit. We used a similar model for the TMR and herbage FA concentrations, but with no repeated measures. Animal related variables (milk FAs, MY, MSY, milk composition, ECM, feed intake, and methane emissions) were averaged and analyzed as one value per cow per week, with cow being considered as the experimental unit. Data were analyzed using generalized linear mixed models (Glimmix procedure), including diet type, week, and their interaction, as fixed effects and block as a random effect. Week was included as the repeated measure. Normality and homogeneity of variances were checked using the univariate procedure and the normal or gamma distribution were including in the model when pertinent. Differences in least squares means in all linear models were investigated using the *t*-test, following Tukey adjustment for multiple comparisons, considering significant differences at *P* < 0.05. The interactions mentioned for each model were removed if they did not tend to be substantial (*P* > 0.1). The results are presented as least square mean ± standard error of the mean (SEM).

## Results

### Feed and Diets

#### Herbage Characteristics

**Figure 2:**
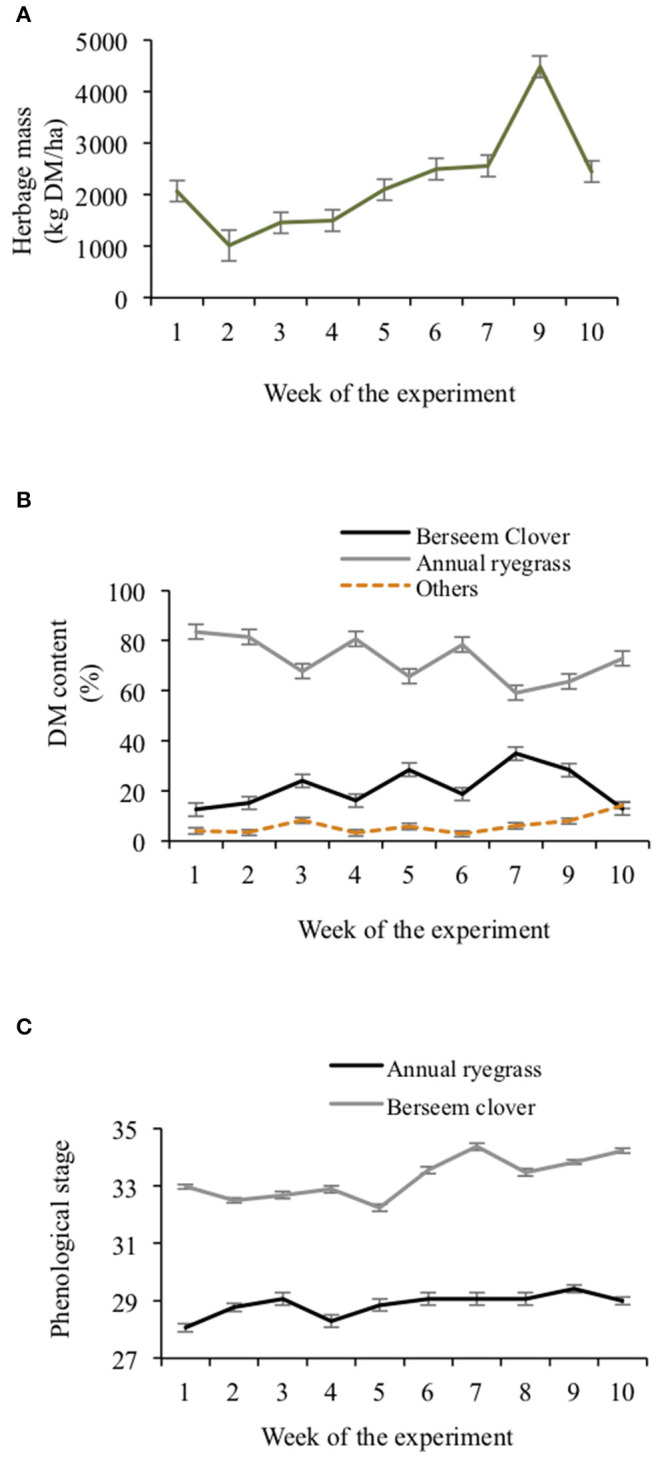
Mixed herbage mass **(A)**, mixed herbage species dry matter content **(B)** and mixed 6 herbage species phenological stage **(C)** throughout the experimental weeks. Error bars represent SE.

Herbage mass had a steady increase with time and was maintained below 2,600 kg DM/ha in most weeks, but in the ninth week it increased to 4,480 kg DM/ha (Figure 2). Proportions of the mixed sward that were berseem clover (*P* < 0.001) and annual ryegrass (*P* < 0.01) changed over time (Figure 2). The mean sward's berseem clover proportion was 21.3% ± 4.30%. The proportion of the sward that was berseem clover was greatest (*P* = 0.02) in week 7 (34.8% ± 3.97%) and the lowest (*P* = 0.01) in the first and the last weeks (12.7 SEM 4.63%). Proportions of the sward that was annual ryegrass trended oppositely to the berseem clover. The proportion of the sward that was other species was <5% for the duration of the experiment. Throughout the experiment, annual ryegrass was in the tillering stage (32.2–34.3) and berseem clover was in stem elongation stage (28.1–29.4) (Figure 2).

#### Diet Composition

Chemical composition of the diets is presented in Table 2. The MIX diet had lower DM (*P* < 0.001), CP (*P* < 0.001), and ADF (*P* < 0.05) concentration than the CON diet, but had greater ME (*P* = 0.03), DM digestibility (*P* < 0.001), cellulose (*P* < 0.001), and ash (*P* < 0.01) concentrations. The chemical composition of the diets forage ingredients: herbage, hay, and silage, is presented in Appendix 1.

**Table 2:** Chemical composition (g/kg DM unless stated otherwise) of the diets offered to the cows during the experimental weeks: a similar to the diet usually offered to the cows (CON) and the experimental diet (MIX).

	**Diet type**			
	**CON**	**MIX**	**SEM**	***P* value**
Dry matter	518	408	5.7	<0.001
Dry matter digestibility (%)	78.6	81.6	0.43	<0.001
Metabolic energy (MJ/kg)	10.96	11.21	0.071	0.03
Crude protein	170	148	2.8	<0.001
Neutral detergent fiber	325	330	6.2	0.52
Acid detergent fiber	175	162	4.7	0.05
Ash	79	109	5.4	<0.01
Cellulose	149	169	2.2	<0.001
Hemicellulose	133	121	5.7	0.16
Lignin	43	41	2.6	0.60
Ether extract	22	22	0.3	0.93

The FA profile of the diets is shown in Table 3. Out of 26 FA found, diets differed in six. The proportions of C16:1 n-3 *trans*, C16:1 n-9 *trans*, and C14:0 was greater (*P* < 0.05) in the MIX diet compared to the CON diet, whereas C15:0, C18:0, and C18:2 n-6 (linoleic acid) were lower. Table 4 shows the FA profile of herbage, silage, and hay. From the same 26 FA, 22 were different in proportion between forage types (*P* < 0.05).

**Table 3:** Fatty acids content (g/100 g of total fatty acids) of the diets offered to the cows during the experimental weeks: a similar to the diet usually offered (CON) and the experimental diet (MIX).

	**Diet**			
**Fatty acid**	**CON**	**MIX**	**SEM**	***P* value**
C14:0	0.39	0.54	0.033	0.02
C15:0	0.20	0.15	0.016	0.05
C16:0	18.6	19.0	1.202	0.82
C16:1 n-3 *trans*	0.35	1.04	0.01	<0.01
C16:1 n-9 *trans*	0.01	0.05	0.006	<0.01
C17:0	0.22	0.18	0.018	0.19
C18:0	2.50	2.03	0.124	0.04
C18:1 n-11 *trans*	0.02	0.03	0.018	0.58
C18:1 n-9 *trans*	0.12	0.05	0.023	0.06
C18:2 n-6	37.64	27.71	1.547	<0.01
C18:3 n-3	5.24	20.18	5.201	0.08
C20:0	0.51	0.44	0.037	0.26
C20:1 n-11 *cis*	1.88	4.93	3.518	0.56
C20:1 n-8 *cis*	0.06	0.07	0.008	0.33
C20:3 n-3	0.01	0.03	0.007	0.20
C20:6 n-2	0.04	0.04	0.003	1
C22:0	0.46	0.51	0.053	0.51
C22:3 n-3	0.25	0.20	0.052	0.52
C22:4 n-6	0.13	0.13	0.035	0.92
C23:0	0.15	0.09	0.026	0.19
C24:0	0.45	0.38	0.06	0.49
C25:0	0.06	0.06	0.016	1
C26:0	0.14	0.22	0.032	0.12
C28:0	0	0.05	0.035	0.36
SFA	24.64	25.97	1.581	0.57
MUFA	31.09	24.12	4.659	0.33
PUFA	43.31	48.28	4.188	0.43

*SFA, saturated fatty acids; MUFA, monounsaturated fatty acids; PUFA, polyunsaturated fatty acids*.

**Table 4:** Fatty acids content (g/100 g of total fatty acids) of the different forage types offered to the cows: mixed herbage (annual ryegrass and berseem clover) included in the experimental diet (MIX) and corn silage and alfalfa hay included in CON diet.

	**Forage type**				
**Fatty acid**	**Mixed herbage**	**Hay**	**Silage**	**SEM**	***P* value**
C14:0	0.80^a^	0.75^a, b^	0.61^b^	0.046	0.04
C15:0	0.21^b^	0.83^a^	0.08^b^	0.041	<0.001
C16:0	20.82^b^	31.77^a^	18.37^b^	0.928	<0.001
C16:1 n-3 *trans*	1.87^a^	1.43^a^	0.27^b^	0.149	<0.001
C16:1 n-9 *trans*	0.11^a^	0.09^b^	0.03^b^	0.016	0.02
C17:0	0.19^b^	0.66^a^	0.19^b^	0.031	<0.001
C18:0	2.00^b^	4.24^a^	2.60^b^	0.165	<0.001
C18:1 n-11 *trans*	0.02	0	0	0.009	0.41
C18:1 n-9 *trans*	0	0	0.04	0.011	0.06
C18:2 n-6	12.01^c^	19.87^b^	45.3^a^	1.01	<0.001
C18:3 n-3	45.94^a^	22.05^b^	7.83^c^	1.694	<0.001
C20:0	0.50^b^	1.12^a^	0.60^b^	0.049	<0.001
C20:1 n-11 *cis*	0.08^b^	0.12^a^	0.14^a^	0.012	0.02
C20:1 n-8 *cis*	0.05^b^	0.04^b^	0.13^a^	0.012	<0.001
C20:3 n-3	0.06^a^	0.05^a^	0.01^b^	0.007	<0.001
C20:6 n-2	0.02	0.03	0.03	0.013	0.85
C22:0	0.93^b^	1.43^a^	0.43^c^	0.059	<0.001
C22:3 n-3	0.06	0.05	0.01	0.007	0.43
C22:4 n-6	0.04	0.05	0.08	0.034	0.72
C23:0	0.17^b^	0.58^a^	0.15^b^	0.033	<0.001
C24:0	0.61^b^	1.32^a^	0.57^b^	0.067	<0.001
C25:0	0.07^b^	0.18^a^	0.12^a,b^	0.017	<0.01
C26:0	0.51^a^	0.56^a^	0.15^b^	0.054	<0.001
C28:0	0.07	0.01	0.02	0.042	0.57
SFA	32.31^b^	47.87^a^	24.69^c^	0.983	<0.001
MUFA	6.68^b^	7.17^b^	20.47^a^	0.560	<0.001
PUFA	58.14^a^	42.03^b^	53.37^a,b^	1.258	<0.001

*SFA, saturated fatty acids; MUFA, monounsaturated fatty acids; PUFA, polyunsaturated fatty acids*.

a, b, c *Means with different superscripts are significantly different (P < 0.05)*.

### Animal Measurements

**Figure 3:**
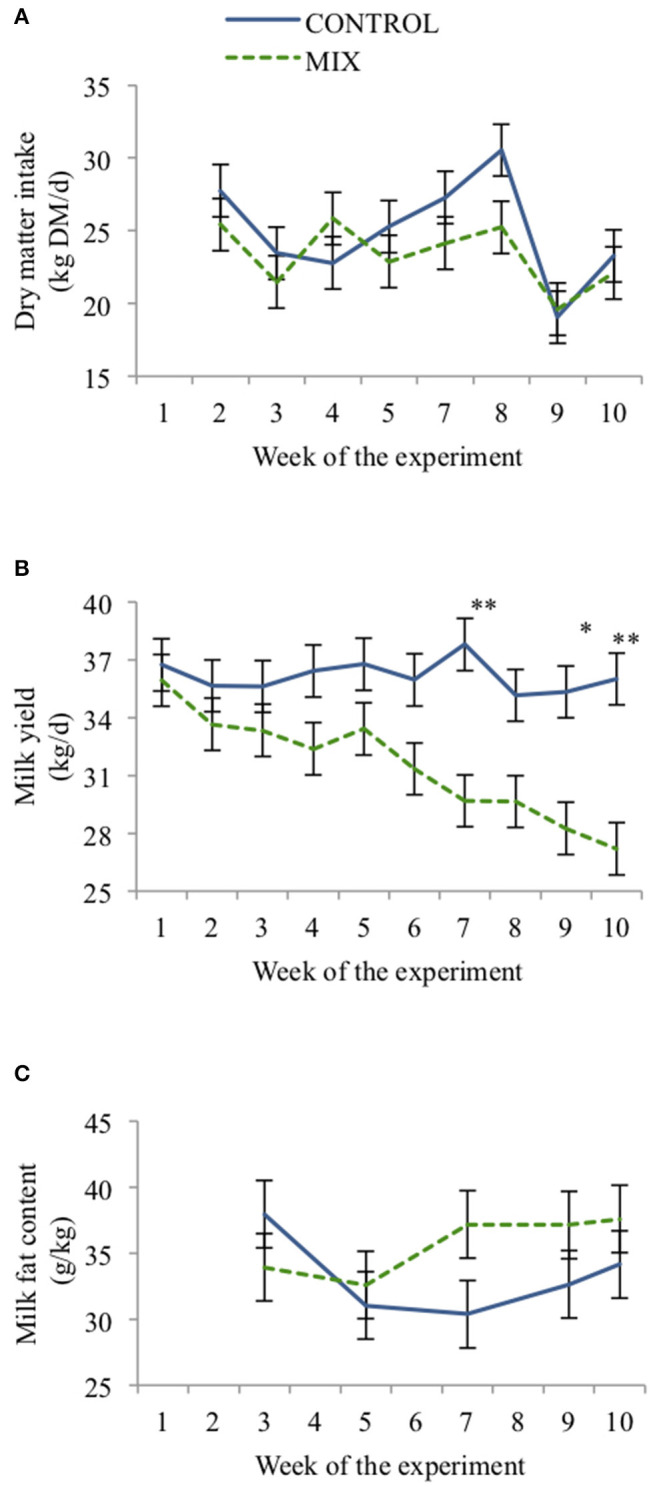
Effect of TMR type (CON = black, MIX = gray) on dairy cow (*n* = 24) daily dry matter intake **(A)**, milk yield **(B)**, and milk fat content **(C)** across the experiment. Error bars represent SE. *P < 0.05, **P < 0.05.

Cows from both diets had similar DMI throughout the experiment (24.1 ± 1.45 kg DM/d, Figure 3), but there was an interaction between weeks and diet type (*P* < 0.001). Both groups reduced DMI from weeks 2 to 3 (*P* < 0.05), but DMI was relatively constant for both groups until week 5 when it started to increase (*P* < 0.05) until week 8 for the CON cows. In the ninth week both groups reduced their DMI (*P* < 0.001), but increased again in the 10th week.

Cow BW and BCS were different between weeks (Table 5), but not between diets.

**Table 5:** Effect of the different diets: a similar to the diet usually offered to the cows (CON) and the experimental diet (MIX) on dairy cows (*n* = 24) performance across the experimental period.

	**Diet type**			***P*** **value**		
	**CON**	**MIX**	**SEM**	**Diet**	**Week**	**Diet × week**
Dry matter intake (kg/d)	24.9	23.3	1.45	0.44	<0.001	<0.001
Milk production (kg/d)						
Yield	36.4	31.9	1.02	<0.01	<0.001	<0.01
Solids yield	2.42	2.10	0.056	<0.001	0.23	0.92
Energy corrected milk	36.2	31.2	1.29	<0.01	0.36	0.75
Milk composition (g/kg)						
Fat	33.2	35.7	2.07	0.41	0.09	0.02
Protein	34.7	34.2	0.82	0.66	<0.001	0.18
Lactose	53.2	52.6	0.39	0.32	0.61	0.60
Milk urea N (mg/dL)	17.5	13.6	1.20	0.03	<0.001	0.20
Body weight (kg)	567	558	129	0.53	<0.001	0.81
Body condition score	3.21	3.14	0.053	0.31	<0.001	0.56

#### Milk

Cows offered the MIX diet had lower MY, MSY, and ECM than CON cows (Table 5). Milk yield (*P* < 0.01) and milk fat concentration (*P* < 0.05) also changed differently for both diets along the experiment (Figures 3A,B). Cows offered the CON diet had a similar MY throughout the experiment, whereas MIX cows showed a reduction over time, especially from week 6 onward. Milk fat concentration from CON cows had a reduction from weeks 3 to 7 (*P* < 0.05), but it increased for MIX cows (*P* < 0.05) from weeks 5 to 7 and afterward was maintained constant through the experiment. The diets did not affect the milk protein or lactose concentrations. Milk urea N was higher for CON cows than for MIX cows.

Milk FA profile is presented in Table 6. From the 43 FA identified, 20 are presented in different proportions between the diets (*P* < 0.05). The proportions of C10:0, C11:0, C14:1, C16:0, C16:1, C18:1 n-10 *trans*, C18:2 n-6 *trans*, C18:2 n-6 *cis*, polyunsaturated (PU):n-6, and the atherogenic and thrombogenic index were greater (*P* < 0.05) for cows on the CON diet, whereas cows offered the MIX diet presented greater (*P* < 0.05) proportions of C13:0, C15:1, C18:0, C18:1 n-9 *cis*, C20:0, C20:5 n-3, C22:2, C24:0, and C24:1 n-9.

**Table 6:** Effect of the different diets: a similar to the diet usually offered to the cows (CON) and the experimental diet (MIX), on milk fatty acids content (%) of dairy cows (*n* = 24).

	**Diet type**			***P*** **value**		
**Fatty acid**	**CON**	**MIX**	**SEM**	**Diet**	**Week**	**Interaction**
C4:0	3.56	3.66	0.067	0.29	<0.001	<0.01
C6:0	2.85	2.92	0.048	0.22	<0.001	0.03
C8:0	2.09	1.98	0.069	0.26	0.76	0.12
C10:0	3.05	2.73	0.123	0.05	<0.001	0.26
C11:0	0.48	0.34	0.023	<0.001	<0.001	0.28
C12:0	4.22	4.23	0.115	0.91	<0.001	0.24
C13:0	0.26	0.43	0.046	<0.01	<0.001	0.03
C14:0	13.84	13.96	0.248	0.70	<0.001	0.25
C14:1	0.79	0.66	0.038	0.03	<0.001	<0.001
C15:0	0.78	0.90	0.051	0.10	0.24	0.06
C15:1	0.50	0.61	0.033	0.03	<0.001	0.75
C16:0	39.77	37.81	0.313	<0.001	0.12	<0.05
C16:1	0.87	0.59	0.034	<0.001	<0.001	0.40
C17:0	0.94	0.89	0.076	0.61	<0.001	<0.001
C17:1	0.43	0.41	0.015	0.25	0.08	0.53
C18:0	6.02	7.68	0.249	<0.001	0.02	<0.001
C18:1 n-10 *trans*	0.21	0.13	0.020	<0.01	<0.001	0.05
C18:1n-11 *trans*	0.25	0.23	0.024	0.42	0.96	0.75
C18:1 n-9 *cis*	13.18	14.06	0.261	<0.01	<0.001	<0.001
C18:2 n-6 *trans*	1.39	1.09	0.047	<0.001	<0.001	0.06
C18:2 n-6 *cis*	1.27	1.06	0.071	0.04	0.32	0.22
C18:2 *cis* 9, *trans* 11	0.41	0.49	0.036	0.14	<0.001	0.23
C18:3 n-6	0.83	0.89	0.037	0.18	<0.001	<0.01
C18:3 n-3	0.80	0.85	0.037	0.29	<0.001	<0.001
C20:0	0.05	0.31	0.037	<0.001	<0.001	<0.01
C20:3 n-6	0.38	0.36	0.027	0.55	<0.001	0.03
C20:4 n-6	0.11	0.09	0.014	0.48	<0.001	0.57
C20:5 n-3	0.01	0.02	0.003	<0.01	0.10	0.17
C22:1 n-9	0.08	0.09	0.032	0.75	0.09	0.21
C22:2	0	0.03	0.005	<0.001	0.51	0.52
C22:6 n-3	0.01	0.02	0.004	0.11	0.22	<0.001
C23:0	0.03	0.06	0.012	0.08	0.08	<0.01
C24:0	0	0.04	0.006	<0.001	0.47	0.47
C24:1 n-9	0.01	0.02	0.003	<0.01	0.05	0.02
SFA	77.94	77.94	0.255	0.99	<0.001	<0.001
MUFA	16.45	16.82	0.234	0.19	<0.001	<0.001
PUFA	5.61	5.24	0.164	0.07	<0.001	0.42
PUFA/SFA	0.07	0.07	0.002	0.06	<0.001	0.76
PU:n-3	1.26	1.24	0.057	0.67	<0.001	0.03
PU:n-6	3.97	3.50	0.121	<0.05	0.06	0.46
n-6/n-3	3.39	3.16	0.148	0.22	<0.001	0.11
Atherogenic index	3.72	3.40	0.085	<0.01	<0.001	<0.001
Thrombogenic index	3.56	3.35	0.072	0.03	<0.001	<0.001

*SFA, saturated fatty acids; MUFA, monounsaturated fatty acids; PUFA, polyunsaturated fatty acids*.

#### Methane

Diet chemical composition, animal measurements, and methane emissions during the period of methane estimation are shown in Table 7. The CON diet had greater DM and CP concentration than the MIX diet but had lower NDF and cellulose concentrations. Cows offered the CON diet had greater MY, MSY, and ECM, but lower (*P* < 0.05) milk fat concentration than MIX cows. In general, there was no effect of diet on methane emissions, methane yield, or methane intensity. However, we note that methane emission was numerically 15% greater and methane yield was numerically 10% greater in the CON cows than the MIX cows, and CON cows emitted 13.6% more methane per kg of milk fat produced than the MIX cows.

**Table 7:** Effect of the control (CON) and the experimental diet (MIX) on diet chemical composition, animal measurements, and methane emissions and efficiency of dairy cows (*n* = 24) during the weeks of methane emission estimations.

	**Diet type**			
	**CON**	**MIX**	**SEM**	***P* value**
**Diet chemical composition**				
Dry matter	523	392	7.0	<0.001
Dry matter digestibility (%)	79.3	81.3	0.73	0.10
Metabolic energy (MJ/kg)	11.09	11.17	0.136	0.68
Crude protein	166	143	3.6	<0.01
Neutral detergent fiber	318	342	6.3	0.03
Acid detergent fiber	172	169	6.5	0.79
Ash	78	109	9.6	0.06
Cellulose	146	173	1.7	<0.001
Hemicellulose	128	132	6.4	0.70
Lignin	44	38	4.0	0.31
Ether extract	22	22	0.5	0.91
**Animal measurements**				
Dry matter intake (kg DM/d)	24.6	24.0	1.84	0.82
Milk yield kg/cow/d	34.3	28.9	1.45	<0.01
Milk solids yield kg/cow/d	2.27	2.02	0.081	0.02
Energy corrected milk (kg/d)	33.6	29.8	1.18	0.01
Milk fat content (g/kg)	29.9	37.2	2.23	0.03
Milk protein content (g/kg)	36.1	35.1	1.25	0.36
Body weight (kg)	577	568	15.0	0.58
**Methane**				
Emission (g/d)	381	332	31.0	0.82
Yield (g/kg DMI)	15.5	13.8	0.79	0.14
**Intensity**				
g/kg ECM	11.5	10.7	0.97	0.43
g/kg of milk fat	368	317	23.9	0.05
g/kg of milk protein	317	319	31.6	0.96
g/kg of body weight	0.66	0.58	0.04	0.26

#### Nitrogen Balance

Table 8 presents data related to the cows' N metabolism: urine ureic N, fecal N concentration, N intake, daily fecal N, daily urine ureic N, and daily milk N were lower (*P* < 0.01) for MIX cows than CON cows. Apparent N absorption was higher for CON cows, but apparent N retention and N use efficiency were similar for both groups. Plasma ureic N concentration was higher for CON cows than MIX cows (12.7 vs. 6.9; SEM: 0.49 mg/dL, *P* < 0.001).

**Table 8:** Effect of the control (CON) and the experimental diet (MIX), on dairy cows (*n* = 24) nitrogen parameters (g/d unless specified otherwise) during the last week of the experiment.

	**Diet type**			
	**CON**	**MIX**	**SEM**	***P* value**
Urine ureic N, (mg/dL)	76.2	38.6	5.85	<0.001
Fecal N, %	2.6	2.3	0.04	<0.001
N intake	684	527	34.3	<0.01
Daily fecal N	239	181	8.7	<0.001
N apparently absorbed	446	347	31.3	0.03
Daily urine ureic N	196	78	15.5	<0.001
Daily milk N	191	149	5.8	<0.001
Apparent N retention^a^	43	111	31.8	0.12
N use efficiency (%)	30.9	30.8	1.93	0.97

a *Calculated by difference (retention = N intake – fecal N – urine N – milk N)*.

## Discussion

Replacing 40% of the alfalfa hay and corn silage in the TMR of dairy cows with a fresh forage mixture did not sustain MY, so we reject our first hypothesis. This difference in MY occurred despite there being no difference in feed intake, implying that the impact on MY was due to differences in the nutritional characteristics of the diets. The diets differed in ME and CP despite being formulated to be isocaloric and isonitrogenous according to our previous information. Nutritional characteristics influence nutrient ingestion and rumen metabolism (3) and consequently the amount and proportion of volatile FA produced during fermentation (38). The CON diet was more abundant in CP and had lower cellulose concentration, which reduces the acetate:propionate ratio in the rumen and enhances protein synthesis and energy source to the cow, thus having positive effects on MY and MSY (39). In contrast, despite the higher DM digestibility and ME of the MIX diet than CON diet, the CP concentration in the MIX diet was lower than the 17% desirable to support MY (18). Although berseem clover in a mature stage can attain 18% CP (7), and the CP concentration of a mixed herbage from the previous year was 16.3% (unpublished data), the CP concentration in the mixed herbage was only 12%, and the resulting CP in the MIX diet was only 14.8%. This was despite our mixed herbage being in the ideal vegetative phenological stage to be harvested. Annual ryegrass was in the tillering stage (20), and berseem clover was in the stem elongation stage (21). During these growth stages, plants have high nutritional value, which means high CP concentration. It is possible that the particularly harsh late-autumn and winter conditions observed in the experimental year (Figure 1) may have reduced the ability of berseem clover to fix N (40), limiting its CP concentration at harvest. Moreover, we included fresh herbage at a rate of 25% in the diet, but berseem clover proportion was low (21.3%), also contributing to low CP concentration and finally no positive effect on milk parameters (41, 42). A mixed herbage with greater berseem clover proportion or an additional source of CP would probably overcome such deficit of the diet, and this warrants further investigation.

Methane yield and methane intensity of both groups were in the lower range of the values reported by Niu et al. (43), which might be ultimately related to the high concentrate proportions of diets tested in this study. Methane yield decreases as the concentrate proportion of the diet increases above 30% (44), and our diets had 54.1% and 49.6% for the CON and the MIX diets, respectively. However, methane emissions, having been reported to increase with increasing proportion of forage in the diet (11), were numerically greater for the CON cows compared to the MIX cows, but not statistically different within the power of our experiment. This means our second hypothesis remains plausible. The diets offered differed in DM, CP, NDF, and cellulose concentrations. The greater CP and lower NDF concentrations of the CON diet were expected to shift fermentation toward propionate production (45), thus reducing substrate availability for methanogenic bacterial activity and methane production. In contrast, the slightly greater DM digestibility of the MIX diet could have induced higher feed intake, thereby increasing passage rate and reduce methane production for MIX cows (46). This must have made up for the numerically lower methane yield and methane intensities from MIX cows than the CON cows. This is similar to Smith et al. (47), who recently reported a numerical reduction in methane emissions when sward white clover proportion was 24%, but this white clover proportion was sufficient to cause a shift toward a less methanogen rumen microbial community. Therefore, it is possible that the low proportion of berseem clover in the herbage and/or the low N concentration of the MIX diet reduced the extent to which the potential for berseem clover to mitigate methanogenesis could be realized.

Nitrogen use efficiency, N intake converted into milk protein N, was not affected by diet, so we reject our third hypothesis. Part of the protein intake by ruminants is broken down into amino acids and then into ammonia in the rumen to be used by bacteria as their primary N source for growth (48). When energy is required in the course of this process, the balance between protein and energy is vital for an efficient microbial synthesis and MY (49). When energy supply is insufficient, the surplus N created passes through the rumen wall to the blood then converted to urea in the liver and finally excreted in milk and urine (50, 51). Increased levels of urea excretion in urine indicate that the additional absorbed N was unavailable to the host animal for growth or production of milk (52), which means overfeeding with protein or low N use efficiency by bacteria in the rumen (48, 50). Milk urea N values and ureic N fraction in blood, urine, and feces of cows eating the CON diet were in excess of baseline values (52, 53), but those cows had higher MY than MIX cows. We expected that the inclusion of fresh forage in the MIX diet would decrease N excretion while maintaining or even increasing MY (3, 54) as legumes can provide a better balance of N and energy intake. There is a linear response between diet CP concentration and milk urea N (53). Therefore, there is a trade-off between milk production, which is promoted by high dietary N, and N use efficiency, which is promoted by low dietary protein. While treatment groups had similar N use efficiency, this tension was reflected in our results with the cows offered the CON diet having greater MY but also greater overall N excretion than those cows offered the MIX diet. The high N loses from cows on the CON diet is worrisome as it represents a poor dietary N utilization. This not only incurs economic costs, but may also represent an important source of environmental pollution (55).

Proportions of FA identified as beneficial to human health were lower in milk from cows fed the CON diet compared to those fed the MIX diet. This link between the FA profile of milk and the inclusion of fresh forages in the diet of dairy cows has been reported previously (56). Some FAs considered as beneficial to human health, such as PUFAs, cannot be synthesized by ruminants but can be supplied by dietary ingredients (57). The MIX diet had a higher proportion of total PUFA, but we noticed no effect on milk. The most abundant PUFA n-3 in milk is linolenic acid (C18:3 n-3; 44), and its proportion was higher in the mixed herbage compared to hay and silage. However, this advantage of the mixed herbage was diluted in the MIX diet and ultimately, no effect of diet was observed on linolenic acid proportion in milk. However, oleic acid (18:1 n-9), a crucial unsaturated FA with a significant role in protecting the human body from cardiovascular diseases (58), was greater in milk produced from MIX cows. Similarly, milk from cows fed the MIX diet had a greater proportion of eicosapentaenoic acid (C20:5 n-3), an essential n-3 PUFA (4). Long-chain omega-6 (n-6) FAs are also crucial for human health, but in excess, they might increase the risk of obesity, type 2 diabetes, and proinflammatory processes (59, 60). Therefore, an elevated ratio of n-6 to n-3 is not desired (60). The CON diet was more abundant in linoleic acid (C18:2 n-6), probably due to the high proportion of this FA in corn silage. However, this proportion was not reflected in milk from the CON cows, plus the ratio of n-6 to n-3 in milk was not affected by diet. While good proportions of different beneficial FA appeared in milk produced from both diets, the index of atherogenicity and thrombogenicity were greater in milk produced from CON cows. Those indices indicate the probability of increasing pathogenic phenomena, such as atheroma and/or thrombus formation, caused by single FA effects (61). Furthermore, some short-chain FAs present in higher proportion in CON milk, such as myristic (C14:0) and palmitic acids (C16:0), have adverse effects on human health due to their relationship with an incidence of atherosclerosis, obesity, and coronary heart diseases (58).

## Conclusion

Replacing 40% of the corn silage and alfalfa hay in the winter diets of dairy cows with a mixed fresh forage resulted in a reduction in MY. This reduction is thought to stem from the concentration of CP being too low in the MIX diet. Despite the reduction in MY, nitrogen use efficiency and methane emissions were unaffected, and milk from cows fed the MIX diet had an FA profile considered to be more beneficial to human health than that of the milk from cows fed the CON diet. The deficit of CP could be overcome either by direct supplementation or increasing the proportion of legume in the mixed herbage (over 20%), which could also positively affect methane and N outcomes.

## Data Availability Statement

The datasets generated for this study are available on request to the corresponding authors.

## Ethics Statement

The study was reviewed and approved by the Scientific Ethics Committee for Animals and Environmental Care of the Pontificia Universidad Católica de Chile (protocol number 160511004).

## Author Contributions

DE-H and DH contributed to the concept of the work and designed the study. DE-H and DLT performed statistical analysis. FCP and DE-H performed the experiment and wrote the manuscript. DLT, LCPMF, DH, PT-M, and SROW contributed to the manuscript. All authors helped with data interpretation and approved the final version of the manuscript.

## Conflict of Interest

The authors declare that the research was conducted in the absence of any commercial or financial relationships that could be construed as a potential conflict of interest.
