# Supplementary material for: Incorporating a Fresh Mixed Annual Ryegrass and Berseem Clover Forage Into the Winter Diet of Dairy Cows Resulted in Reduced Milk Yield, but Reduced Nitrogen Excretion and Reduced Methane Yield
Source: Front Vet Sci. 2020 Nov 20;7:576944. doi: 10.3389/fvets.2020.576944 (PMC7714779; doi:10.3389/fvets.2020.576944)
Supplement: Supplementary file 1 [file Data_Sheet_1.docx]

**APPENDIX 1.** Chemical composition (g/kg DM unless stated otherwise) of the diets forage ingredients.

|  | Forage type | | |  |  |
| --- | --- | --- | --- | --- | --- |
|  | Mixed  herbage^1^ | Alfalfa hay | Maize silage | SEM | P-value |
| Dry matter | 193^c^ | 889^a^ | 378^b^ | 7.4 | <0.001 |
| Dry matter digestibility (%) | 85.7^a^ | 62.0^c^ | 79.2^b^ | 0.814 | <0.001 |
| Metabolic energy (Mcal/kg) | 2.76^a^ | 2.05^b^ | 2.73^a^ | 0.025 | <0.001 |
| Crude protein | 120^a^ | 150^a^ | 70^b^ | 5.0 | <0.001 |
| Neutral detergent fiber | 398^b^ | 499^a^ | 335^c^ | 12.0 | <0.001 |
| Acid detergent fiber | 195^b^ | 363^a^ | 174^b^ | 8.8 | <0.001 |
| Ash | 89^b^ | 127^a^ | 60^c^ | 5.2 | <0.001 |
| Cellulose | 201^a^ | 137^c^ | 158^b^ | 5.2 | <0.001 |
| Hemicellulose | 276^a^ | 160^b^ | 149^a^ | 9.1 | <0.001 |
| Lignin | 35^b^ | 87^a^ | 25^b^ | 3.2 | <0.001 |
| Ether extract | 1.52^b^ | 1.00^c^ | 2.39^a^ | 0.08 | <0.001 |

^1^Annual ryegrass and berseem clover.
